# Supplementary material for: Surface Microbiome Profiling of Dental Elevators Using Third‐Generation Sequencing: Implications for Infection Control in Dental Practice
Source: Microbiologyopen. 2025 Dec 8;14(6):e70178. doi: 10.1002/mbo3.70178 (PMC12685768; doi:10.1002/mbo3.70178)
Supplement: Supplementary file 1 — customer_backup. [file MBO3-14-e70178-s001.zip › customer_backup/customer_backup/taxa_summary/krona/groups/treat/M13.Krona.html]

Javascript must be enabled to view this page.

members
magnitude
magnitudeUnassigned

M13.krona

67516

67516

21

18

18

6

6

6

6

6

6

6

6

6

0

0

0

0

3

3

3

3

0

0

0

3

0

0

0

0

0

1

1

1

1

0

0

0

0

0

0

0

0

1

1

11

7

0

0

0

0

0

0

0

0

0

0

0

0

0

0

0

0

0

7

0

0

0

0

0

0

0

0

1

0

0

1

1

5

5

5

1

1

1

0

0

0

0

0

0

0

0

0

0

0

0

4

0

0

0

0

0

0

0

4

0

0

0

1

1

1

0

0

0

1

1

1

2

2

2

212

7

7

0

0

0

7

3

3

0

3

0

3

0

0

1

1

37

1

1

1

1

33

0

0

0

1

1

1

1

0

0

0

0

1

1

31

3

2

1

1

1

3

3

0

16

2

2

3

9

2

2

6

1

5

3

3

3

3

0

11

11

11

9

9

2

2

145

145

113

0

0

1

1

1

1

1

1

4

4

1

1

105

105

0

1

1

1

0

0

0

0

0

31

31

1

24

0

6

0

0

12

12

12

0

0

12

12

0

0

0

0

0

0

0

0

0

0

0

0

0

0

0

0

0

0

0

0

0

0

0

0

0

0

2

2

2

2

2

2

0

0

0

0

0

0

0

0

0

0

0

0

0

0

2

2

2

0

0

0

2

2

2

0

0

0

0

0

0

0

0

0

8

0

0

0

0

0

0

0

0

0

0

0

0

0

1

1

1

1

1

7

7

7

0

0

0

7

2

0

5

0

0

0

0

0

0

0

31

1

1

0

0

0

1

1

1

4

3

2

2

2

0

0

0

1

1

1

1

1

1

0

1

25

3

3

1

0

1

0

2

0

2

0

0

0

0

0

0

0

0

0

0

0

0

0

0

0

22

0

0

0

0

0

0

0

0

2

0

0

2

2

0

0

16

0

0

0

0

0

0

16

16

4

4

1

3

0

0

0

0

0

0

0

0

0

0

0

0

0

1

1

1

1

1

13767

659

0

0

0

0

0

0

0

0

0

0

0

0

0

0

0

0

0

0

0

0

0

0

0

0

0

0

0

0

0

0

0

0

0

1

0

0

0

1

0

0

0

0

0

0

1

1

0

0

0

0

0

0

0

0

0

0

0

0

0

0

0

0

0

0

0

0

0

259

259

0

0

1

0

0

0

0

1

0

0

0

0

0

0

0

0

0

0

0

0

0

0

1

0

0

0

0

1

0

0

0

0

1

0

1

0

0

0

0

0

255

0

0

0

0

0

0

99

0

0

0

0

0

0

0

0

0

0

0

0

0

155

0

0

0

0

1

0

0

0

0

1

0

0

0

1

0

0

0

0

0

0

0

0

0

106

1

0

0

1

0

1

0

0

0

0

0

0

0

0

0

0

0

0

0

0

0

1

0

0

0

0

0

0

0

0

0

0

0

0

1

1

0

0

0

0

0

0

0

0

0

0

0

0

0

0

0

17

0

0

0

0

1

0

1

5

5

0

0

0

0

3

3

1

0

1

0

0

0

0

3

0

2

0

1

0

0

0

0

0

4

0

0

0

0

2

0

0

0

0

0

1

1

0

0

0

0

0

0

0

0

0

0

0

0

0

0

0

0

0

0

0

0

0

0

0

0

0

4

0

0

0

0

4

3

1

0

0

0

0

0

0

0

4

3

0

3

1

0

1

28

0

0

0

0

0

1

1

0

0

0

0

0

0

0

0

0

0

0

27

0

27

0

0

0

0

51

1

1

0

0

0

0

0

0

0

0

0

0

0

0

0

0

0

0

0

0

0

0

0

0

0

0

0

0

0

48

0

48

0

0

0

0

0

0

0

0

2

2

0

0

0

0

0

0

1

0

0

0

0

0

1

0

0

0

0

0

0

0

0

0

0

0

0

0

0

0

1

1

0

0

0

0

0

0

7

7

0

0

0

0

7

2

2

3

0

0

0

0

0

0

0

0

0

0

0

0

0

0

0

0

0

0

0

0

0

0

0

1

0

0

0

1

1

1

0

0

0

0

0

0

0

221

221

0

0

1

0

1

220

220

0

0

0

0

0

0

0

0

0

0

0

0

0

0

0

0

0

0

0

0

0

0

0

0

0

0

0

0

0

0

0

0

0

0

0

0

0

0

0

0

0

0

0

0

0

0

0

0

0

0

0

0

0

0

0

0

0

63

63

0

0

0

0

1

1

0

0

1

1

0

0

0

0

0

0

0

0

0

0

0

1

1

1

1

0

0

0

0

0

0

0

0

0

0

0

0

0

59

0

1

58

0

0

0

0

0

0

0

0

13108

0

0

0

0

0

1

1

0

0

1

1

0

0

0

0

0

0

0

0

12072

1

1

1

0

0

0

0

0

0

0

0

0

0

0

0

0

0

0

0

0

0

0

0

0

0

0

0

0

0

0

0

0

0

0

0

4865

0

0

0

0

4863

4863

1

0

0

1

0

1

0

0

0

0

0

1

0

0

0

0

0

0

0

0

0

0

1

0

0

1

1

0

0

0

0

0

0

0

0

0

0

0

0

0

0

92

0

0

0

0

0

0

0

0

0

0

2

0

0

2

90

90

0

0

0

0

0

0

0

0

0

0

0

0

0

0

0

7109

0

0

0

0

0

0

0

0

1

1

0

0

0

0

0

0

1

0

1

0

0

1273

0

1273

0

0

0

0

0

468

0

468

0

0

0

1

0

1

5365

5365

0

0

4

0

0

4

0

4

259

0

0

0

0

0

0

0

0

259

257

219

38

0

0

2

2

0

0

0

0

146

135

135

135

11

11

11

0

0

0

0

0

0

0

0

0

4

4

4

1

3

0

0

0

0

0

0

0

0

0

0

236

0

0

0

219

0

0

0

0

0

0

0

1

1

218

213

0

0

5

0

0

0

0

0

0

0

0

0

0

0

0

0

0

0

1

1

1

0

0

0

10

10

0

0

7

3

5

1

0

0

1

0

0

0

4

4

0

0

0

0

0

0

1

1

1

0

0

0

0

0

0

0

0

1

1

0

0

1

1

0

0

0

0

0

1

1

1

1

10

5

5

5

5

1

1

2

2

2

1

1

1

1

1

1

344

0

0

0

0

0

0

0

0

0

0

0

0

0

0

1

1

1

104

53

53

46

46

5

5

0

0

1

1

0

1

1

1

1

0

0

0

2

2

2

235

36

36

0

0

199

199

0

0

0

0

0

0

0

0

0

0

0

0

0

0

0

0

0

0

0

0

0

0

0

0

0

0

0

0

0

0

0

0

0

0

0

0

0

0

0

0

0

0

0

0

0

0

0

0

0

0

0

0

0

0

0

0

0

0

0

0

0

0

0

0

0

0

0

0

0

0

0

0

0

0

0

0

0

0

0

0

0

0

0

0

0

0

3

3

3

3

0

0

0

0

0

0

0

2

2

2

2

0

0

0

0

0

0

0

20

20

0

0

0

0

0

0

0

0

0

0

0

0

20

6

0

14

0

0

0

0

0

0

0

0

0

0

7

1

1

1

2

2

2

4

4

4

1

1

1

0

1

0

0

0

0

0

0

0

23

14

14

0

0

0

14

0

0

0

0

0

0

0

0

14

1

13

0

0

0

0

0

0

0

0

9

0

0

0

0

0

0

0

0

0

0

0

0

0

0

0

0

0

7

0

0

0

0

0

0

0

0

0

0

0

0

0

0

0

0

0

0

1

1

1

6

0

0

0

6

6

0

0

0

0

0

0

0

0

0

0

0

0

2

2

2

0

0

2

0

0

0

0

0

0

0

0

0

0

0

0

0

0

0

0

0

0

0

0

0

0

0

0

0

0

0

0

0

0

0

0

0

0

0

0

0

1216

1

1

1

1

1

1207

126

0

0

0

1

0

0

0

1

0

1

0

0

0

0

0

0

0

0

0

0

0

0

0

0

0

0

0

0

0

0

0

0

0

0

0

0

0

0

0

0

0

0

0

0

0

0

0

0

0

0

0

0

0

0

0

0

0

0

125

125

125

0

0

0

0

0

122

0

0

0

91

1

1

0

0

0

0

0

0

0

0

0

0

0

0

0

0

0

0

0

0

0

0

0

0

0

0

0

0

0

0

0

1

1

0

0

0

0

0

0

89

89

0

0

0

0

0

0

0

0

0

0

0

0

0

31

0

0

31

31

0

0

0

0

883

2

2

2

0

0

0

0

0

0

0

0

137

0

0

3

3

26

26

108

108

5

0

0

0

0

0

0

0

0

0

0

0

0

0

5

0

2

2

0

0

0

0

1

0

0

0

0

0

6

0

0

0

0

6

2

0

4

2

1

1

1

1

0

0

0

66

66

0

0

0

22

25

0

0

0

0

12

2

0

2

0

0

0

3

143

5

5

125

0

125

13

3

2

3

1

0

4

505

0

0

0

0

0

0

8

0

0

8

0

0

0

0

0

0

0

0

161

0

0

0

0

24

0

0

0

0

0

0

0

0

0

0

0

137

336

0

0

0

0

69

267

0

0

0

0

0

0

0

0

0

0

0

0

0

3

3

3

13

13

12

1

0

0

0

0

1

1

1

0

0

0

0

0

0

0

0

0

0

0

0

0

0

0

0

0

0

0

0

0

0

0

0

0

0

0

0

0

76

0

0

0

0

0

0

0

0

0

0

0

1

0

0

1

1

0

0

0

0

0

0

1

0

0

0

0

1

0

1

0

0

0

0

0

0

0

0

11

11

11

0

0

0

0

0

0

63

0

0

0

0

0

0

0

0

0

0

0

0

0

0

0

0

0

63

0

0

0

0

0

0

0

0

0

63

0

0

0

0

0

0

0

0

3

3

3

0

0

3

3

0

0

0

0

0

0

0

0

5

5

2

2

0

2

3

1

1

2

2

0

0

0

0

4

4

1

1

0

0

1

1

0

0

0

0

2

0

0

0

2

0

0

2

2

0

0

0

0

0

0

0

0

0

0

0

0

0

0

0

1

1

1

1

0

0

0

0

0

0

0

0

0

0

0

0

0

0

0

5

5

5

5

4

4

1

1

10998

370

370

0

0

0

342

300

66

0

0

234

42

0

0

42

0

28

0

0

0

0

0

0

0

0

0

2

2

17

0

17

0

0

0

0

0

5

0

5

0

4

0

3

1

0

0

0

0

0

0

0

46

23

23

1

1

6

6

3

3

0

0

0

0

11

11

2

2

0

0

0

0

1

0

0

0

0

0

0

1

1

1

0

0

0

0

0

0

0

0

0

0

0

0

0

0

0

0

0

0

0

0

0

0

0

0

0

0

0

0

0

0

0

0

22

3

0

0

0

1

1

0

2

0

2

0

2

2

0

0

2

16

0

0

0

15

0

15

1

1

0

1

1

1

0

0

0

0

13

10

0

0

0

1

1

0

0

0

0

0

1

0

0

0

0

0

0

0

0

0

0

0

0

0

0

0

0

9

1

1

0

0

8

8

0

0

0

0

3

2

2

1

1

0

1

1

1

0

0

0

0

10569

0

0

0

0

0

0

0

0

20

20

20

20

0

0

0

0

0

0

0

0

24

24

17

17

0

0

0

0

0

0

0

0

0

7

7

0

0

0

0

0

0

0

0

0

0

0

0

0

0

0

0

0

0

0

0

0

0

0

0

0

0

0

384

384

384

0

368

0

16

0

0

0

0

0

0

0

0

0

0

0

0

0

0

0

0

0

0

24

24

0

0

0

24

0

18

1

0

0

5

0

0

0

0

2

1

0

0

1

1

0

0

0

0

0

0

0

0

0

0

0

0

0

0

0

0

0

0

1

0

0

1

1

0

0

0

0

0

0

0

0

0

0

0

0

0

0

0

8963

8963

1377

1370

7

0

7586

229

0

98

0

44

198

6870

0

0

16

30

101

0

0

0

0

0

0

0

0

0

0

0

0

0

0

0

0

0

0

0

0

158

152

1

1

0

0

0

0

0

0

0

0

0

0

0

0

0

0

151

0

151

0

0

0

6

0

0

0

0

0

0

6

0

0

0

0

0

0

0

0

0

0

0

0

0

0

6

0

0

0

0

0

0

0

0

0

0

0

0

0

0

0

5

5

0

0

0

0

0

0

0

0

0

5

5

0

0

0

0

0

0

0

0

0

0

0

0

0

0

0

0

0

0

989

957

0

0

955

955

0

0

0

0

0

0

0

0

1

0

1

0

0

0

0

0

0

0

1

1

0

0

0

0

0

0

0

0

0

0

0

0

0

0

0

0

0

0

0

0

2

2

1

0

1

0

0

29

0

0

0

0

0

0

1

1

0

1

1

0

0

0

0

0

0

0

0

0

0

0

0

1

1

2

0

0

2

0

0

0

0

24

24

0

0

0

0

0

1

0

0

1

1

0

0

0

0

0

0

0

0

2

2

2

2

0

0

0

0

0

0

0

0

0

0

0

0

0

0

0

0

0

0

0

0

0

0

0

0

0

0

2

2

0

0

0

0

0

0

0

0

0

0

0

0

27

27

27

27

27

27

2560

2560

2560

156

0

0

2

2

0

0

154

0

154

2404

2404

1432

0

0

0

972

0

0

0

0

700

700

700

1

1

0

1

0

0

0

697

697

0

697

0

0

0

0

0

0

0

2

2

2

0

0

0

0

0

0

0

0

0

0

0

0

0

0

0

0

0

0

0

0

0

0

0

0

0

0

0

0

0

0

0

0

0

0

0

0

106

7

3

3

3

3

4

1

1

1

0

0

0

0

0

0

0

3

0

0

0

0

0

0

3

3

0

0

0

0

0

0

0

0

0

0

0

0

0

0

0

0

0

0

0

0

0

0

0

0

0

0

0

0

0

0

0

0

0

0

0

0

0

0

0

0

0

0

0

0

0

0

0

96

96

96

9

9

87

0

8

0

0

0

0

0

79

2

2

2

2

2

1

1

0

0

0

1

1

1

0

0

0

0

0

0

0

147

0

0

0

0

0

0

0

0

0

0

0

0

0

0

0

0

0

0

0

0

0

0

0

0

0

0

0

0

0

0

0

0

0

0

0

0

0

0

0

0

0

0

0

0

0

0

0

0

0

0

0

0

0

0

0

0

0

0

0

0

0

0

0

1

1

1

1

1

146

146

0

0

0

0

0

0

0

0

0

0

0

0

0

0

0

0

0

146

0

0

0

0

0

0

0

146

146

0

0

0

0

0

0

0

0

0

0

1

1

1

0

0

0

1

1

1

0

0

0

0

0

0

0

0

0

0

0

0

0

0

0

0

0

0

0

0

0

0

0

0

0

0

0

0

0

0

0

0

0

0

0

0

0

0

0

0

0

0

0

0

0

0

0

0

0

0

0

0

0

0

0

0

0

0

0

0

0

0

0

0

0

0

0

0

0

0

17

17

17

0

0

0

0

0

17

0

0

0

17

0

0

0

0

17

0

0

1

1

1

1

1

1

0

0

37466

2603

2

2

0

0

0

0

0

2

0

1

0

0

0

1

0

0

0

0

0

0

3

0

0

0

0

0

0

3

3

3

0

0

10

10

10

0

10

0

1736

1735

5

5

0

0

3

3

0

0

0

16

4

12

1

1

0

71

70

0

1

0

0

1

1

0

2

2

0

0

213

213

0

0

0

0

0

0

0

0

7

0

0

7

0

0

8

0

0

0

0

1

0

0

0

0

5

0

0

1

1

0

0

0

0

0

0

0

0

0

0

4

0

4

0

0

1

0

1

0

57

28

0

0

29

0

0

149

0

0

0

0

149

0

0

0

0

0

0

85

3

0

1

80

1

30

30

0

0

814

814

0

0

0

0

0

1

0

1

0

0

1

1

1

1

47

0

1

39

7

1

1

0

0

18

18

0

0

0

0

0

0

0

0

0

0

7

0

7

0

0

0

192

1

93

0

18

1

22

9

41

0

4

1

2

0

0

0

1

1

1

0

0

0

0

403

7

0

0

0

0

0

0

0

0

0

0

0

0

0

0

0

0

6

0

0

0

0

6

1

1

0

0

0

396

0

0

0

0

1

1

0

0

0

0

0

0

395

395

0

0

0

0

0

0

0

0

0

0

0

0

0

0

0

172

172

0

0

0

0

2

0

0

0

0

0

0

2

170

170

143

0

0

0

0

0

0

140

140

140

0

0

0

3

3

3

0

0

0

0

0

0

0

0

0

100

71

2

0

2

0

0

3

3

0

0

0

0

0

0

0

0

0

0

0

0

2

2

0

0

2

2

0

0

0

3

0

1

0

2

0

2

0

1

0

0

0

0

0

1

0

1

1

0

5

5

3

0

3

48

0

0

45

3

6

6

6

0

0

0

4

0

0

0

3

3

0

0

0

1

1

0

0

0

0

0

0

0

0

0

0

0

19

5

0

1

0

0

3

1

1

1

0

0

1

1

0

0

0

0

0

3

2

0

0

1

9

9

0

0

0

0

0

0

0

0

0

34

0

0

0

0

0

0

34

34

34

0

0

0

0

0

0

19245

0

0

0

0

773

3

0

0

0

0

0

0

1

0

1

0

0

0

0

0

0

2

2

0

0

0

0

0

0

770

0

0

1

1

0

0

0

769

0

0

0

0

0

0

0

1

0

0

0

207

2

0

0

236

323

0

0

0

0

0

0

0

0

0

0

0

0

0

1264

30

0

0

1

1

9

9

12

7

0

0

0

5

0

0

0

3

3

3

1

2

2

2

1234

151

151

362

362

5

1

4

3

0

3

638

638

0

0

0

0

0

7

7

5

0

5

0

0

59

59

0

0

2

2

2

2

0

0

0

0

0

0

0

0

322

99

1

1

3

3

95

91

4

223

223

219

0

4

1

1

1

1

0

93

93

0

0

0

0

92

91

0

0

0

0

0

0

0

0

0

0

0

0

0

0

0

0

0

0

1

0

0

0

0

0

0

0

0

1

1

0

0

0

0

0

0

0

0

0

0

0

0

0

0

0

0

0

1

1

1

1

0

16791

3

3

3

0

244

0

0

0

0

244

244

0

0

0

0

13950

13950

8

1633

81

0

1051

0

2

341

2938

7709

185

0

2

0

0

0

2

2

0

2

2583

37

37

0

0

8

6

1

1

31

0

0

31

1240

0

328

90

0

0

822

0

0

780

779

0

0

0

0

1

2

2

472

0

0

472

4

3

1

9

9

0

0

0

0

0

0

9

0

0

9

9

0

0

0

0

0

0

0

0

0

0

0

0

0

0

0

0

0

0

0

0

0

0

0

0

0

0

0

0

0

0

0

0

0

0

15618

0

0

0

0

0

0

15618

0

0

0

1052

0

0

0

0

0

0

1

1

0

1051

0

807

150

94

0

0

0

0

0

14565

14540

14540

0

0

0

25

0

25

0

0

0

0

0

1

1

1

0

0

0

0

0

2

0

0

0

0

0

0

0

0

0

0

0

0

0

0

0

0

0

0

0

0

0

0

0

0

0

0

0

0

0

0

0

0

0

0

0

0

0

0

0

0

2

0

0

0

0

2

2

2

0

2

0

0

0

0

0

0

0

0

0

0

0

0

0

0

0

0

0

0

0

0

0

0

0

0

0

0

0

0

0

0

0

0

0

0

0

185

185

185

185

185

0

185

0

0

0

0

0

0

0

1

1

1

1

1

1

0

0

0

0

0

0

0

0

0

0

0

0

0

0

0

0

0

0

0

0

0

0

0

0

0

0

0

0

0

0

0

0

0

0

0

0

0

0

0

0

0

0

0

0

0

0

0

0

0

0

0

0

0

0

0

0

0

0
